# CPSF6 links alternative polyadenylation to metabolism adaption in hepatocellular carcinoma progression

**DOI:** 10.1186/s13046-021-01884-z

**Published:** 2021-03-01

**Authors:** Sheng Tan, Ming Zhang, Xinglong Shi, Keshuo Ding, Qiang Zhao, Qianying Guo, Hao Wang, Zhengsheng Wu, Yani Kang, Tao Zhu, Jielin Sun, Xiaodong Zhao

**Affiliations:** 1 Key Laboratory of Systems Biomedicine (Ministry of Education), Shanghai Center for Systems Biomedicine, Shanghai Jiao Tong University, Shanghai, 200240 China; 2 Department of Integrated Traditional Chinese and Western Medicine, Shanghai Chest Hospital, Shanghai Jiao Tong University, Shanghai, 200030 China; 3 School of Biomedical Engineering, Shanghai Jiao Tong University, Shanghai, 200240 China; 4 Department of Pathology, School of Basic Medicine, Anhui Medical University, Hefei, 230032 Anhui China; 5 Hefei National Laboratory for Physical Sciences at Microscale, the CAS Key Laboratory of Innate Immunity and Chronic Disease, School of Life Sciences, University of Science and Technology of China, Hefei, 230027 Anhui China

**Keywords:** Alternative polyadenylation, Hepatocellular carcinoma, Metabolism, CPSF6, NQO1

## Abstract

**Background:**

Alternative polyadenylation (APA) is an important mechanism of gene expression regulation through generation of RNA isoforms with distinct 3′ termini. Increasing evidence has revealed that APA is actively involved in development and disease, including hepatocellular carcinoma (HCC). However, how APA functions in tumor formation and progression remains elusive. In this study, we investigated the role of cleavage factor I (CFIm) subunit CPSF6 in human hepatocellular carcinoma (HCC).

**Methods:**

Expression levels of CPSF6 in clinical tissues and cell lines were determined by qRT-PCR and western blot. Functional assays, including the cell number, MTT, colony formation and transwell, were used to determine the oncogenic role of CPSF6 in HCC. Animal experiments were used to determine the role of CPSF6 in HCC tumorigenicity in vivo. Deep sequencing-based 3 T-seq was used to profile the transcriptome-wide APA sites in both HCC cells and CPSF6 knockdown HCC cells. The function of CPSF6-affected target *NQO1* with distinct 3′UTRs was characterized by metabolism assays.

**Results:**

We observed CPSF6 was upregulated in HCC and the high expression of CPSF6 was associated with poor prognosis in patients. Overexpression of CPSF6 promoted proliferation, migration and invasion of HCC cells in vitro and in vivo. Transcriptome-wide APA profiling analysis indicated that high expression of CPSF6 promoted the favorable usage of the proximal poly(A) site in the 3′UTR of *NQO1*. We demonstrated CPSF6-induced tumorigenic activities were mediated by the *NQO1* isoform with short 3′UTR. Furthermore, we found that CPSF6 induced metabolic alterations in liver cells through NQO1.

**Conclusion:**

CPSF6 plays a critical role in HCC progression by upregulating NQO1 expression through APA. These findings provide evidence to demonstrate that APA of *NQO1* contributes to HCC progression and may have implications for developing new therapeutic strategy against this disease.

**Supplementary Information:**

The online version contains supplementary material available at 10.1186/s13046-021-01884-z.

## Background

Hepatocellular carcinoma (HCC), the main form of liver cancer, is one of the most common cancer types and the third leading cause of cancer-related mortality worldwide [1, 2]. Many risk factors, such as hepatitis B virus (HBV) and hepatitis C virus (HCV) infections, autoimmune hepatitis, cirrhosis, alcohol abuse and toxic chemical exposures, may play a role in HCC progression [3, 4]. Moreover, genetic alterations in the *TERT*, *TP53*, and *CTNNB1* loci have been detected in early stage or advanced HCC [5]. Despite significant progress, the overall survival rate of HCC patients is still unsatisfactory, mainly owing to their high rates of recurrence [6]. Therefore, the molecular mechanisms of HCC remain to be investigated and new therapeutic targets need to be identified.

Alternative polyadenylation (APA) is a post-transcriptional mechanism to generate distinct 3′-untranslated regions (UTRs) of a given gene, increasing transcriptome complexity [7]. The 3′UTRs contain regulatory elements for miRNA and RNA-binding protein binding sites, which allows for regulation of those gene products and provides an important layer of gene expression regulation [8–11]. APA potentially affects mRNA stability, translation efficiency and subcellular localization of the transcript isoforms. About 70% of human genes have multiple poly(A) sites. The selective usage of APA sites has been characterized in various biological contexts, including cellular proliferation, differentiation, neuron activation and cancer [7, 12–16].

An interesting topic of cancer biology is that APA contributes to proto-oncogene activation and thereby promotes oncogenic transformation [17]. The preferential usage of APA sites has been observed in various types of cancer, such as breast, kidney, colon, liver and lung tumors [18–20]. Moreover, APA patterns could be used as biomarkers for cancer diagnosis and prognosis [21]. These observations suggest APA is actively associated with the initiation and progression of cancer. However, the mechanistic links between APA and tumorigenicity remain elusive.

A number of factors have been reported to regulate APA in global or gene-specific manner, among which are RNA 3′-end-processing factors [7]. Some APA regulatory factors are functionally involved in tumor formation. For example, upregulation of CFIm25 in glioblastoma cells suppresses the tumorigenic properties and inhibits tumor growth [22]. In contrast, activation of CSTF2 was observed to enhance the oncogenic activities in urothelial carcinoma of the bladder [23]. These studies suggest the complicated biological consequence of APA factors on tumorigenicity.

We have developed a deep sequencing-based approach 3 T-seq for APA site profiling [24]. Recently, we have reported that CFIm25 (encoded by *Nudt21*) exerted suppressive effect on liver cancer cells [25]. The polyadenylation complex cleavage factor I (CFIm), one of four core components of APA machinery, is composed of two small subunits CFIm25 and two large subunits CFIm68 (also known as CPSF6) and CFIm59 (also known as CPSF7) [26, 27]. CFIm plays a key regulator of cleavage and polyadenylation site choice during APA through its binding to 5′-UGUA-3′ elements localized in the 3’UTR for a huge number of pre-mRNAs [28, 29]. CPSF6 is a component of the CFIm complex that functions as an activator of the pre-mRNA cleavage and polyadenylation processing required for the maturation of pre-mRNA into functional mRNAs [26, 29–31]. Although CPSF6 was reported to be involved in tumor progression in cancer [32], the underlying mechanisms are yet to be completely characterized. In this study, we investigated the role of CPSF6 in HCC. We showed that CPSF6 expression was significantly increased in HCC tissues. CPSF6 knockdown obviously inhibits the proliferation, migration and invasion ability of liver cancer cells Huh-7 and HepG2 in vitro and in vivo. Transcriptome-wide APA profiling analysis indicated that high expression of CPSF6 promoted the favorable usage of the proximal poly(A) site in the 3′UTR of a number of genes, including *NQO1*. We demonstrated CPSF6-induced tumorigenic activities were mediated by the *NQO1* isoform with short 3′UTR. Furthermore, we found that CPSF6 induced metabolic alterations in liver cells through NQO1.

## Methods

### Cells and culture conditions

Human immortalized liver cell line (HL-7702) and liver cancer cell lines (Huh-7, HepG2, SK-HEP-1, PLC/PRF/5, Hep3B) were purchased from Shanghai Cell Bank (Shanghai, China). The cells were cultured in Dulbecco’s modified Eagle’s medium (DMEM, Gibco, Carlsbad, CA, USA) supplemented with 10% fetal bovine serum (FBS, Gibco, Carlsbad, CA, USA), 1% penicillin–streptomycin (Sangon, Shanghai, China), and 2 mM glutamine (Invitrogen, Carlsbad, CA, USA) at 37 °C in a 5% CO_2_ humidified atmosphere.

### Histopathological analysis

Human liver tissues were obtained from the First Affiliated Hospital of Anhui Medical University (Hefei, China). The study was approved by the Biomedical Ethics Committee of Anhui Medical University and informed consent was obtained from each patient. For IHC staining of clinical samples and xenograft tumors, tissue samples were first fixed in formalin and performed following the manufacturer’s instruction. IHC labeling intensity was reviewed and scored by two independent pathologists. The mouse lung tissues were fixed with paraformaldehyde (PFA, 4%) for 12 h, then dehydrated with ethanol and embedded in paraffin. Paraffin-embedded mouse lung tissue was sectioned and then stained with hematoxylin and eosin. Tissue samples for RNA extraction were quickly frozen in liquid nitrogen after resection and kept in RNAlater (CAT #AM7021, Invitrogen, Carlsbad, CA, USA). Total RNA was extracted using Trizol (CAT #15596026, Invitrogen, Carlsbad, CA, USA) following the manufacturer’s instruction.

### Protein extraction and western blot analysis

Cells or tissues were lysed in ice-cold RIPA buffer (50 mM Tris, 150 mM NaCl, 1.0% Triton, 1 mM EDTA, 0.1% SDS) supplemented with protease (CAT #11697498001, Roche, Basel, Switzerland) and phosphatase inhibitor cocktail (CAT #4693132001, Roche, Basel, Switzerland). The protein concentrations were determined by the BCA Assay using BCA Assay protein Kit (Sangon, Shanghai, China). Soluble protein was separated by 10% SDS-PAGE and transfer onto PVDF membranes (Millipore, Billerica, MA, USA). The membrane was incubated in blocking buffer (1xPBST with 1% BSA) for 1 h at room temperature. The membrane was then blotted with the diluted primary antibodies: anti-CPSF6 (1:1000, CAT #ab175237, Abcam), anti-NQO1 (1:1000, CAT #11451–1-AP, Proteintech) and anti-β-actin (1:5000, CAT #A5316, Sigma). Immunoreactive protein was visualized using ECL start Western Blotting Substrate (GE Healthcare Life Sciences, USA) and image quant LAS 4000 mini (GE Healthcare Life Sciences, USA).

### Reverse transcription and quantitative PCR analysis

Total RNA was extracted using TRIzol (CAT #15596026, Invitrogen, Carlsbad, CA, USA) and reversely transcribed using RevertAid™ First Strand cDNA Synthesis Kit (CAT #K1621, ThermoFisher, USA) according to the manufacturer’s instructions. The qRT-PCR reactions were performed using SYBR-GREEN (Applied Biosystems) according to the manufacturer’s instructions. Gene-specific primers for qRT-PCR analysis were as follows: *CPSF6* (F: GGAGCAGCACCAAATGTTGTC, R: CTCCCAAAGAATGAACTGCTTC); *NQO1* (F: CTGATCGTACTGGCTCACTCAG, R: GCAGGATACTGAAAGTTCGCAG); *GAPDH* (F: CACAGTCCATGCCATCACTG, R: CTTGGCAGCGCCAGTAGAG). *GAPDH* was used as an internal control.

### Generation of stable cell lines

The lentiviral vectors pSIN-GFP-puro-CPSF6 and pSIN-GFP-puro-NQO1 were constructed for CPSF6 and NQO1 expression, respectively. The pSIN-GFP-puro was purchased from Genecopoeia. The lentiviral vectors pLKO.1-puro-shRNA-CPSF6 and pLKO.1-puro-shRNA-NQO1 were constructed for CPSF6 and NQO1 knockdown, respectively. The pLKO.1-puro was obtained from Genecopoeia. The 293 T cells were used for the lentiviral production and supernatant was collected at 48 h and 72 h after transfection. The viruses were filtered using 0.45 μm filters and appropriate amounts of viruses were used to infect liver cells. Finally, cells were maintained in the presence of puromycin (2 μg/ml) for 1–2 weeks.

### Luciferase assays

NQO1-luciferase reporter construct (psiCHECK2, Promega, USA) contained the short or the long 3′UTR of the human *NQO1* gene. Liver cells were transfected with NQO1-luciferase reporter vector in 24-well plates (100 ng/well) using Lipofectamine 3000 (Invitrogen, Carlsbad, CA, USA). After transfection for 48 h, cells were harvested and assayed using dual luciferase assay according to the manufacturer’s protocol (Promega, USA). Firefly luciferase read-outs were normalized to Renilla luciferase read-outs. To ensure the reporter with long 3′UTR was expressed, the proximal PAS signals were mutated (AATAAA was mutated to ACAAAC) for these reporters, which contains the long 3′UTR of *NQO1*.

### MTT assay

Liver cells were plated in 96-well plates (1000–3000 cells/well). 3–5 days after seeding, methyl thiazol tetrazolium (MTT) was added into each well (5 mg/mL) for 1–2 h. Then, the media was removed and each well was washed using PBS. DMSO was added into each well and the absorbance was measured at 570 nm.

### Colony formation assays

Liver cells were seeded into 6-well plates (100–300 cells/well) and cultured for 2–3 weeks. At the end points of the experiments, cells were fixed with 4% methanol, stained with crystal violet (0.1%), and photographed.

### Migration and invasion assays

Liver cells were resuspended in serum-free medium. For the migration assays, cells (5–10 × 10^4^ /100 μl) were seeded onto the chamber without Matrigel. For the invasion assays, 10–20 × 10^4^ cells (5–10 × 10^4^ /100 μl) were seeded onto the chamber with 100 μl of Matrigel. The bottom chamber was prepared using 800 μl of DMEM medium (supplemented with 10% FBS) as a chemoattractant. After 24–48 h of incubation, the cells on the outer surface were washed by PBS and fixed with 4% paraformaldehyde, stained in a dye solution containing 0.1% crystal violet for visualization.

### Glucose uptake and lactate measurements assay

The glucose uptake in HL-7702 or Huh-7 cells (2 × 10^4^) was measured using the commercially available kit Glucose Uptake-Glo™ Assay (CAT #ab136955, Abcam, UK) according to the manufacturer’s instructions. HL-7702 or Huh-7 cells (2 × 10^4^) were plated in 96-well plates and lactate production was measured using the commercially available kit (CAT #600450, Cayman, USA) according to the manufacturer’s instructions.

### Xenograft model in vivo

The design and protocol of in vivo experiments were approved by the Institutional Animal Care and Use Committee, Shanghai Jiao Tong University. For tumorigenicity assay, liver cells were resuspended in PBS/Matrigel Matrix mix at 1:1 ratio. 5 × 10^6^ cells in 100 μL solution were injected into the flank of 4–6 weeks old athymic male nude mice. Tumor volumes were calculated as follows: Volume = ½ (L × W^2^), where L is the length and W is the width. For the lung colonization assay, cells were resuspended in PBS and injected into the lateral tail vein of 4–6 weeks old NOD/SCID mice (5 × 10^5^ cells/animal).

### 3 T-seq analysis

3 T-seq analysis was performed as we described previously [24]. Briefly, M280 beads were coated with Bio-oligo dT (20) then incubated with 50 μg total RNA. The cDNA was synthesized and released by *Gsu I* digestion. The 3′UTR fragments were ligated to the Illumina sequencing adaptors and subjected to deep sequencing. The qualified reads were mapped to UCSC human reference genome (hg19) with bowtie2 (version 2.3.3.1) [33]. The 3′UTR switching for each gene in the cell lines was detected by linear trend test (the FDR-adjusted *p*-value < 0.05) [34]. The accession number of raw data is E-MTAB-9458, and available in the ArrayExpress database (http://www.ebi.ac.uk/arrayexpress).

## Results

### CPSF6 is upregulated in HCC and high expression of CPSF6 is associated with poor prognosis in HCC patients

**Fig. 1:**
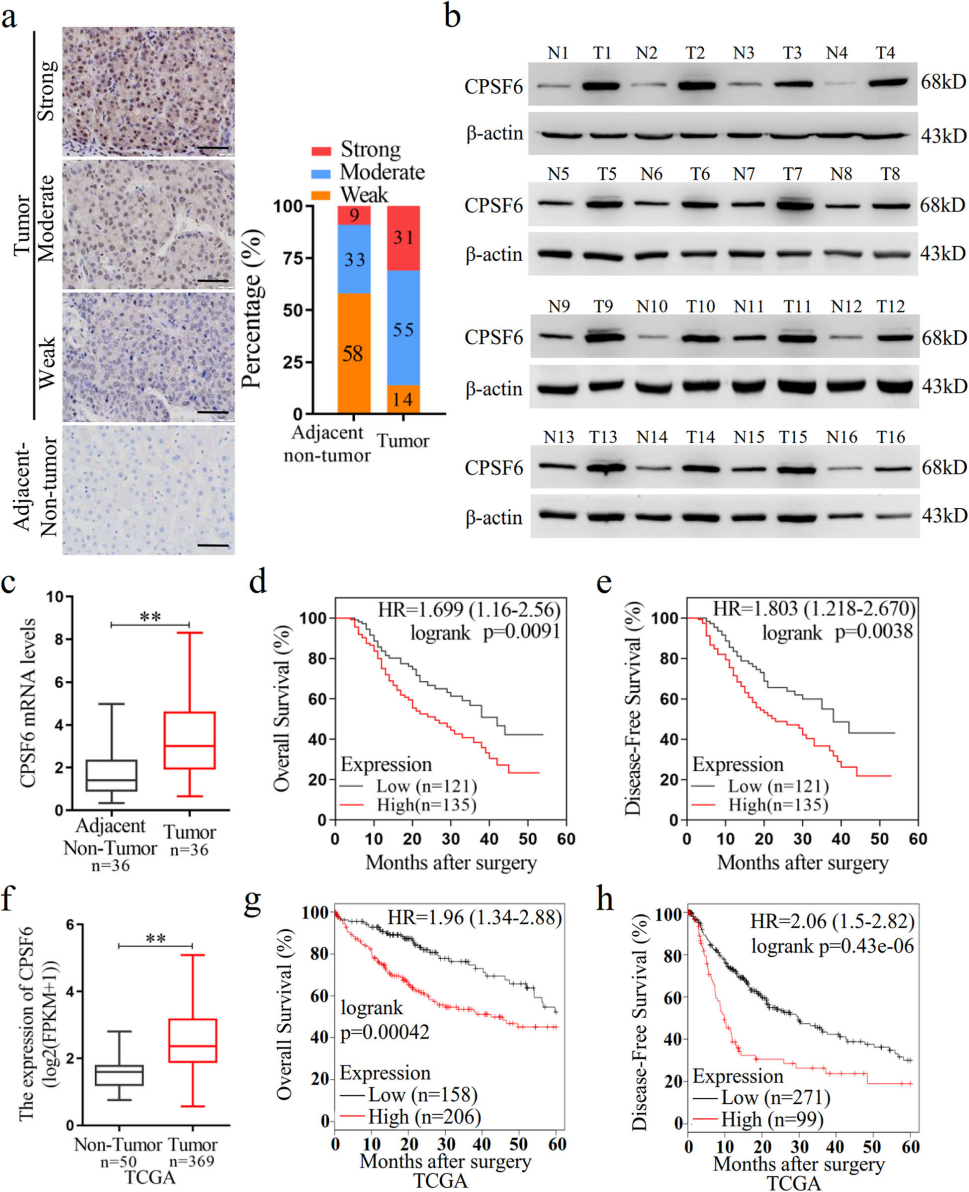
CPSF6 is upregulated and predicts poor prognosis in HCC. **a** IHC staining of CPSF6 in surrounding non-tumor and HCC tissues. Percentage of CPSF6 IHC was shown in bar graph. Scale bar, 50 μm. **b** Western blot assay for detecting CPSF6 expression in 16 paired surrounding non-tumor (N) and HCC tumor (T) tissues. β-actin was used as internal control. **c** qRT-PCR analysis of *CPSF6* mRNA in 36 paired surrounding non-tumor (N) and HCC tumor (T) tissues. *GAPDH* was used as internal control. **d**, **e** Kaplan–Meier analysis of the correlation of CPSF6 protein expression with OS (d) and DFS (e). The expression level of CPSF6 protein was detected by immunohistochemistry. **f** qRT-PCR analysis of *CPSF6* mRNA in TCGA liver cancer samples. g,h Kaplan–Meier analysis of OS (**g**) and DFS (**h**) data from TCGA liver cancer data containing 364 patients. The data of g and f can be obtained through online website (http://kmplot.com/analysis/).**p < 0.001, Student’s t-test

We firstly examined the CPSF6 expression in HCC patients. We collected HCC tissues (*n* = 159) and the paired non-tumor tissues (n = 159) and performed immunohistochemical (IHC) assay. CPSF6 staining was scored as a strong or moderate expression in 31 and 55% of tumor tissues, as compared with 9 and 33% in corresponding adjacent non-tumor liver tissues (Fig. 1a). The elevated expression of CPSF6 was also observed in 33 out of 36 HCC tissues by western blot assay when compared with the adjacent non-tumor counterparts (Fig. 1b and Supplementary Fig. S1). Consistent with the detected protein abundance, we observed the elevated mRNA level of *CPSF6* in HCC tissues by real-time quantitative PCR (Fig. 1c). These data indicated that CPSF6 was upregulated in HCC. The patients with high CPSF6 expression (*n* = 135) had shorter overall survival (OS) (*p* = 0.0091) and disease-free survival (DFS) (*p* = 0.0038) than the patients with low CPSF6 expression (*n* = 121) (Fig. 1d, e). To understand the clinical implications of the increased expression, we examined the correlation of CPSF6 with the clinicopathologic features of HCC. The higher CPSF6 expression was observed to be associated with tumor size (*p* < 0.001), advanced tumor-node metastasis (TNM) stage (p < 0.001), venous invasion (p < 0.001) and distant metastasis (p < 0.001) of the HCC patients (Supplementary Table S1).

We extended our analysis by examining the expression of *CPSF6* with publicly available human clinical data in The Cancer Genome Atlas (TCGA) database. We found that the expression level of *CPSF6* was significantly increased in HCC patients (Fig. 1f). Similarly, we observed that the patients with high *CPSF6* expression also had shorter OS (*P* = 0.00042) and DFS (*P* = 0.000043) (Fig. 1g, h). Together, these observations suggest that CPSF6 is upregulated in HCC and high CPSF6 expression is associated with poor clinical outcomes in the HCC patients.

### CPSF6 promotes HCC cell growth in vitro and in vivo

**Fig. 2:**
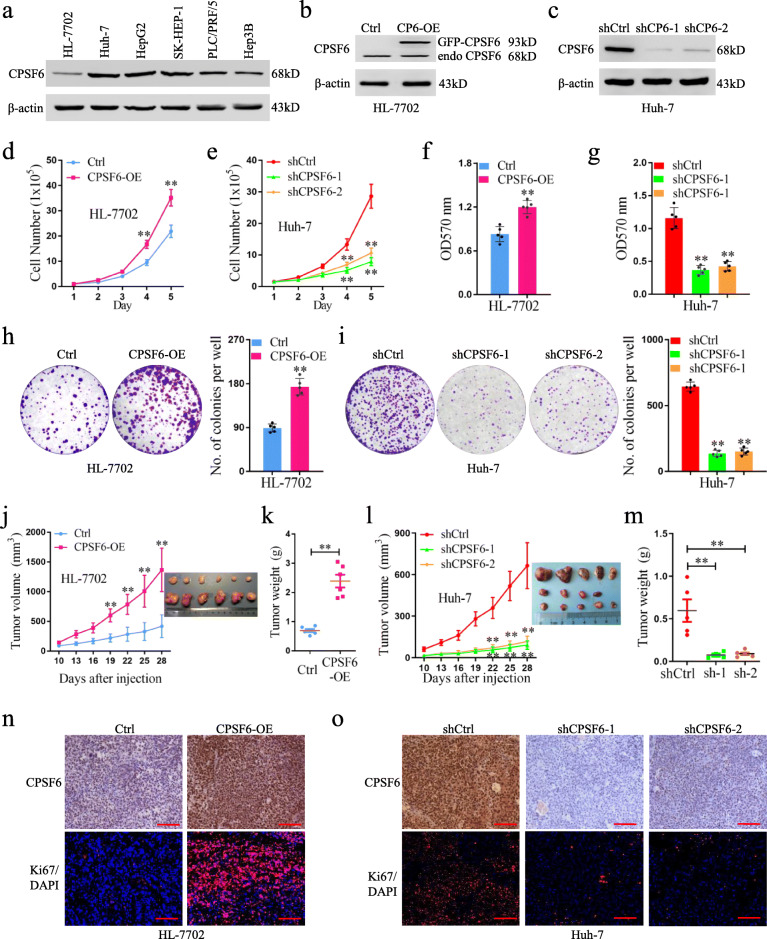
CPSF6 accelerates the growth of HCC cells in vitro and in vivo. **a** The protein level of CPSF6 in different cell lines was assessed by western blotting. β-actin was used as internal control. **b** The protein level of CPSF6 in the CPSF6-overexpressing (CPSF6-OE) HL-7702 was assessed by western blotting. β-actin was used as internal control. **c** CPSF6 knockdown efficiency in Huh-7 with two CPSF6 specific shRNAs (shCPSF6–1 and shCPSF6–2) was examined by western blotting. β-actin was used as internal control. **d** Proliferation curves of the CPSF6-overexpressing HL-7702 cells and the control (Ctrl) were shown. **e** Proliferation curves of the CPSF6 knockdown Huh-7 cells and the control (shCtrl) were shown. **f** The cell viability of HL-7702 cells with CPSF6 overexpression and the control was examined by MTT assay. **g** The cell viability of CPSF6 knockdown Huh-7 cells and Huh-7 cells (the control) was examined by MTT assay. **h**,**i** Colony formation analysis of the indicated cell lines. **j** HL-7702 xenograft tumor growth with or without overexpression of CPSF6. **k** The weight of HL-7702 tumors at the end point was shown. **l** Huh-7 xenograft tumor growth with or without CPSF6 knockdown. **m** The weight of Huh-7 tumors at the end point was shown. **n**, **o** IHC analysis of CPSF6 and Ki67 expression in tumors. Scale bars, 100 μm. **p < 0.001, Student’s t test

We next examined the protein expression of CPSF6 in non-tumor liver cell line HL-7702 and human HCC cell lines (Huh-7, HepG2, SK-HEP-1, PLC/PRF/5 and Hep3B). Compared with HL-7702, higher level of CPSF6 was observed in four HCC cell lines (Huh-7, HepG2, SK-HEP-1 and PLC/PRF/5) (Fig. 2a). To explore the function of CPSF6 in HCC, CPSF6 was ectopically expressed in HL-7702 and repressed with short hairpin RNAs (shRNAs) in Huh-7 and HepG2, respectively (Fig. 2b, c and Supplementary Fig. S2A). Compared with HL-7702 cells (the control), overexpression of CPSF6 in HL-7702 significantly increased cell proliferation (Fig. 2d), viability (Fig. 2f), and generated more and larger colonies in vitro (Fig. 2h). In contrast, CPSF6 knockdown in Huh-7 and HepG2 significantly reduced cell proliferation (Fig. 2e and Supplementary Fig. S2B), viability (Fig. 2g and Supplementary Fig. S2C) and clonogenicity in vitro (Fig. 2i and Supplementary Fig. S2D). To understand CPSF6 role in vivo, HL-7702 cells with overexpression of CPSF6 were injected subcutaneously into nude mice. We observed a significant increase in both tumor growth and weight (Fig. 2j, k); whereas a significant reduction in both tumor growth and weight was observed in CPSF6 knockdown tumors (Fig. 2l, m). Similarly, the alterations in abundance of Ki67 protein were found in CPSF6-overexpressing tumors (Fig. 2n) and CPSF6 knockdown tumors (Fig. 2o). Collectively, our results demonstrated that enforced expression of CPSF6 significantly promoted tumorgenicity in vitro and in vivo.

### CPSF6 promotes HCC cell migration and invasion in vitro and metastasis in vivo

**Fig. 3:**
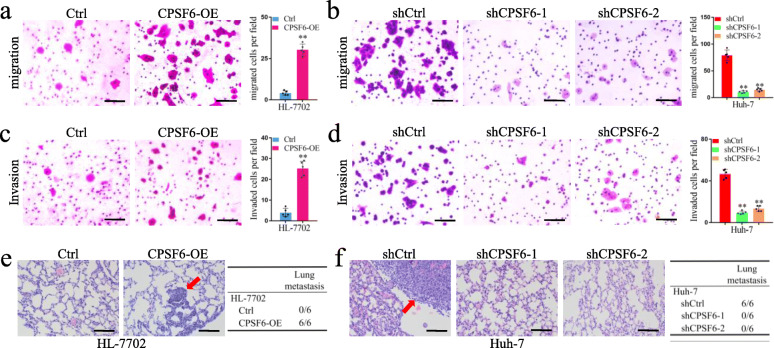
CPSF6 promotes migration, invasion and metastasis of HCC cells. **a** Effect of CPSF6 overexpression on HL-7702 cell migration. **b** Effect of CPSF6 knockdown on Huh-7 cell migration. **c** Effect of CPSF6 overexpression on HL-7702 cell invasion. **d** Effect of CPSF6 knockdown on Huh-7 cell invasion. Representative pictures of H&E staining of lungs and incidence of lung metastasis from mice inoculated with (**e**) HL-7702 and (**f**) Huh-7 cells. Red arrows indicated the lung metastases. Results represented mean ± SD using bar graph. Scale bars, 100 μm. **p < 0.001, Student’s t test

Considering the clinical relevance between CPSF6 and metastasis, we examined whether CPSF6 affects the migration and invasion of HCC cells. As shown in Fig. 3a, forced expression of CPSF6 significantly promoted cell migration capacity in HL-7702 cells, while CPSF6 knockdown significantly inhibited migration of Huh-7 and HepG2 cells (Fig. 3b and Supplementary Fig. S3A). The similar phenotype of cell invasion was observed in the CPSF6-overexpressing HL-7702 cells (Fig. 3c) or the CPSF6 knockdown Huh-7 and HepG2 cells (Fig. 3d and Supplementary Fig. S3B). These results indicated that CPSF6 promoted migration and invasion of HCC cells in vitro. We next injected hepatic cells (HL-7702-Ctrl, HL-7702-CPSF6-OE, Huh-7-shCtrl and Huh-7-shCPSF6) into the tail vein of nude mice (*n* = 6 for each group) and examined the metastatic nodules in lungs. The frequency of lung metastasis from the lungs in HL-7702-CPSF6-OE group was significantly higher when compared with the HL-7702-Ctrl group at one month after injection (Fig. 3e). Meanwhile, we observed that the frequency of lung metastasis was decreased in Huh-7-shCPSF6 group as compared with Huh-7-shCtrl group (Fig. 3f). These observations demonstrated that CPSF6 knockdown suppresses metastasis of HCC cells in vivo.

### CPSF6 modulates the widespread 3′UTR alteration in HCC cells

**Fig. 4:**
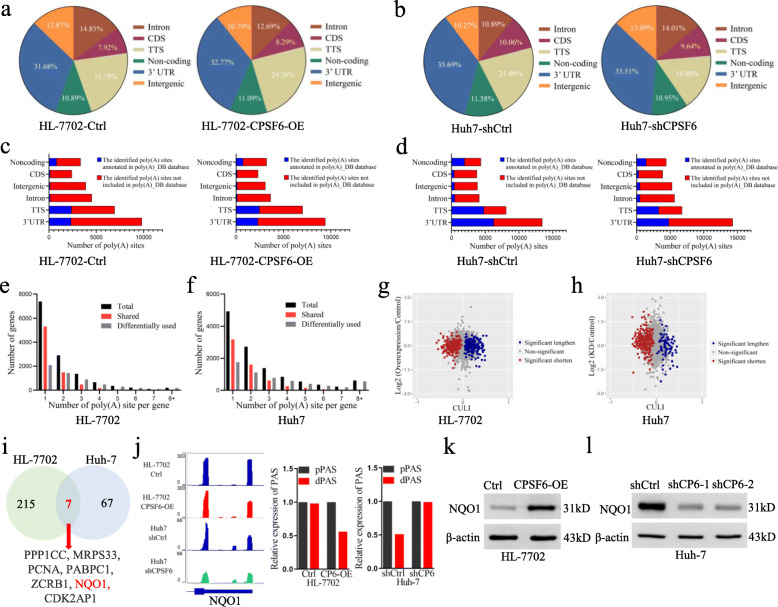
Characteristics of 3 T-seq data. **a**, **b** Genomic locations of qualified 3 T-seq reads mapped to the reference genome. **c**, **d** Genomic distribution of the poly(A) sites. **e**, **f** The statistics of genes with various number of detected poly(A) sites. **g**, **h** Scatterplot of CULI for measuring the 3′UTR alteration in CPSF6-overexpressing or CPSF6 knockdown cells when compared with the corresponding control cells (false discovery rate (FDR) = 0.05). **i** CPSF6-modulated 7 candidate genes. These genes had the shortened 3′UTR in CPSF6-overexpressing HL-7702 cells and the lengthened 3′UTR in CPSF6 knockdown Huh-7 cells. **j** CPSF6-induced APA shift of *NQO1*. Left, Integrative Genomics Viewer (IGV) genome browser exhibited the poly(A) site usage of *NQO1* 3′UTR. Right, histogram showed the relative expression of the isoform with distal polyadenylation site (dPAS) relative to the one with proximal PAS (pPAS). **k**, **l** The protein levels of NQO1 in the CPSF6-overexpressing HL-7702 cells (**k**) and the CPSF6 knockdown Huh-7 cells (**l**) were assessed by western blotting. β-actin was used as internal control

As a member of APA machinery, CPSF6 causes the oncogenic activities described above. We thus hypothesized that CPSF6 functions as an oncogene, at least in part, by influencing APA and 3′UTR. To test this hypothesis, we performed APA site profiling analysis in CPSF6-overexpressing HL-7702 cells, CPSF6 knockdown Huh-7 cells and the corresponding control cells with 3 T-seq we reported previously [24]. A total of 73.1 million reads were obtained, of which 87.7% were uniquely mapped to the reference genome (Supplementary Table S2). After filtering internal priming events, we found most of the qualified reads were mapped to the annotated transcription termination sites (TTSs) or 3′UTRs (Fig. 4a, b), yielding 26,946 poly(A) sites (Supplementary Table S2).

Using the snowball clustering method [34], we found the majority of identified APA sites in each 3 T-seq library were located at the 3′ terminus. In particular, 34–59% of these poly(A) sites were mapped to TTSs and 24–47% to the 3′UTR regions in polyA-DB database [35], respectively. About 30% of these poly(A) sites have been annotated in the polyA-DB database, and thus the remaining sites are putatively novel (Fig. 4c, d). In addition, 28% of genes harbor three or more poly(A) sites (Fig. 4e, f). We adopted the cancer 3′UTR length index (CULI) [34] to quantitatively measure the 3′UTR alteration. Comparing with HL-7702, we identified 433 genes with altered 3′UTR in CPSF6-overexpressing HL-7702 cells, 51.3% of which showed a shift from distal to proximal poly(A) site usage and thus possessed shortened 3′UTRs (Fig. 4g and Supplementary Table S2). Meanwhile, we also identified 74 genes with lengthened 3′UTR in CPSF6 knockdown Huh-7 cells (Fig. 4h and Supplementary Table S3).

To explore how CPSF6-modulated APA contributes to tumorigenicity, we examined the genes with 3′UTR alteration simultaneously influenced under both CPSF6-overexpressing and knockdown conditions, yielding seven candidates (Fig. 4i and Supplementary Table S4). The association between HCC and the metabolic alterations has been recently reported [36, 37]. Interestingly, among seven CPSF6 responsive genes we found the metabolism regulator *NQO1* (Fig. 4i). Furthermore, overexpression of CPSF6 induced the preferential usage of the proximal poly(A) site in *NQO1* 3’UTR; while CPSF6 knockdown caused the shift of polyadenylation from the proximal to the distal (Fig. 4j). Furthermore, western blotting results indicated that CPSF6 positively regulates NQO1, i.e., NQO1 expression increased under the condition of CPSF6 overexpression and decreased in the case of CPSF6 knockdown. (Fig. 4k, l).

### NQO1 short 3’UTR isoform enhances the oncogenic and metastatic capacities of liver cells both in vitro and in vivo

**Fig. 5:**
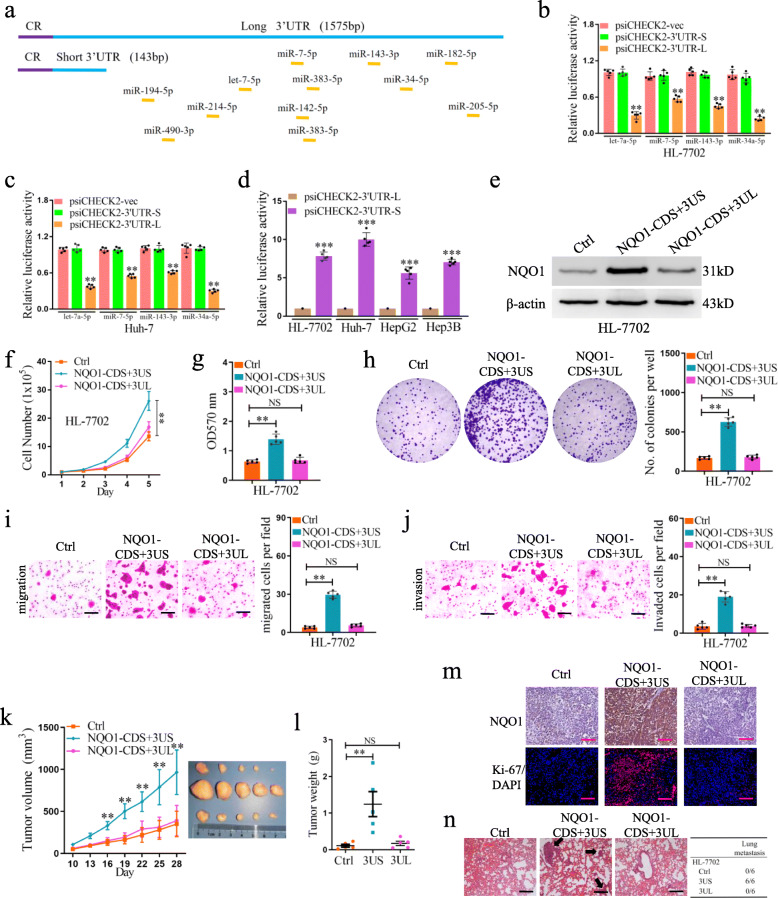
The *NQO1* short 3’UTR isoform has oncogenic function and increases aggressiveness in liver cells. **a** Schematic illustration of the *NQO1* isoform with long or short 3’UTR. Positions of the binding sites of miRNA were indicated by yellow horizontal lines. The activity of short 3’UTR (3’UTR-S) and long 3’UTR (3’UTR-L) of *NQO1* after enforced expression of the specific miRNAs was examined using luciferase reporter assay in HL-7702 (**b**) and Huh-7 (**c**) cells. **d** Luciferase expression from a reporter containing the short 3’UTR of *NQO1*, as compared to that from the reporter containing the long 3’UTR of *NQO1* in 4 liver cell lines. **e** The protein levels of NQO1 in HL-7702 cells stably transfected with *NQO1*-overexpressing plasmids were assessed by western blotting. β-actin was used as internal control. **f** Proliferation curves of HL-7702 cells stably transfected with NQO1-overexpressing plasmids or control (Ctrl) were shown. **g** The cell viability of HL-7702-NQO1-OE and HL-7702-Ctrl cells was examined by MTT assay. **h** Colony formation analysis of HL-7702-NQO1-OE and HL-7702-Ctrl cells. **i** Transwell analysis of the migration ability of HL-7702 cells. **j** Transwell analysis of the invasion ability of HL-7702 cells. **k** HL-7702 xenograft tumor growth with or without overexpression of different *NQO1* isoforms. **l** The weight of HL-7702 tumors at the end point was shown. **m** IHC analysis of NQO1 and Ki67 expression in tumors. Scale bars, 100 μm. **n** Representative pictures of H&E staining of lungs and incidence of lung metastasis from mice inoculated with HL-7702 cells. Black arrows indicate the lung metastases. **p < 0.001, Student’s t test

We performed the prediction analysis of miRNA binding site with online tools TargetSan (http://www.targetscan.org/vert_72/) and starBase V2.0 (http://starbase.sysu.edu.cn/). Compared with the transcript isoform containing short 3′UTR, *NQO1* transcript isoform with long 3′UTR contains multiple potential miRNA binding sites (Fig. 5a). We selected several predicted miRNAs, all of which were reported to have cancer suppression function [38–41]. The effect of the selected miRNAs on translation activity of *NQO1* 3’UTRs was examined by luciferase activity assay. We observed that the let-7a-5p and other three miRNAs had a significant inhibitory effect on the translation activity of the *NQO1* isoform with long 3’UTR, and no significant effect on the isoform with short 3’UTR was observed (Fig. 5b, c). The translation activity of the short 3’UTR is 5.6- to 10.1- fold higher than that of the long 3’UTR (Fig. 5d). As expected, the NOQ1 protein level of the mRNA isoform with short 3′UTR was higher than that with long 3′UTR (Fig. 5e).

To understand the function of distinct *NQO1* transcript isoforms in liver cells, the *NQO1* isoforms with short and long 3′UTRs were transfected into HL-7702 cells, respectively. The results showed that enforced expression of the isoform with short 3′UTR dramatically increased cell growth (Fig. 5f, g), colony formation (Fig. 5h), migration (Fig. 5i) and invasion (Fig. 5j), when compared with that of vector control and ectopic expression of isoform with long 3′UTR. Furthermore, we observed that enforced expression of the short isoform substantially enhanced the activities of subcutaneous tumor formation (Fig. 5k) and increased the tumor weight (Fig. 5l) in mice. The expression of NQO1 and Ki67 was increased in tumors of mice injected with *NQO1* short isoform-expressing HL-7702 cells; while no significant change was observed in the tumors injected with *NQO1* long isoform-expressing HL-7702 cells (Fig. 5m). We next injected HL-7702 cells with *NOQ1* isoforms into the tail vein of nude mice (*n* = 6 for each group) and examined the metastatic nodules in lungs. The results showed that enforced expression of the short 3’UTR isoform of *NQO1* enhanced lung metastasis when compared with that of either ectopic expression of long 3’UTR isoform or the control cells (Fig. 5n).

### The oncogenic effect of CPSF6 is mediated by NQO1

**Fig. 6:**
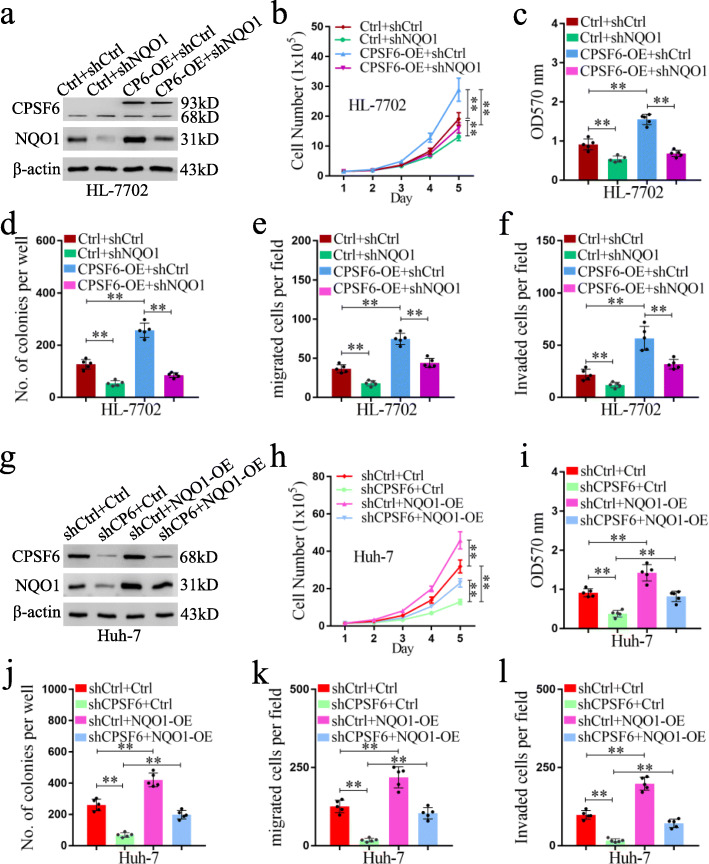
Function of CPSF6 is NQO1-dependent. **a** Western blot analysis of the efficiency of NQO1 knockdown and overexpression of CPSF6 in HL-7702 cells. **b** Proliferation curves of HL-7702 cells were shown. **c** The cell viability of HL-7702 cells was examined by MTT assay. **d** Colony formation analysis of HL-7702 cells. **e** Transwell analysis of cell migration of HL-7702. **f** Transwell analysis of the cell invasion of HL-7702. **g** Western blot analysis of the efficiency of NQO1 overexpression (NQO1-OE) and CPSF6 knockdown in Huh-7. **h** Proliferation curves of Huh-7 cells were shown. **i** The cell viability of Huh-7 cells was examined by MTT assay. **j** Colony formation analysis of Huh-7 cells. **k** Transwell analysis of the cell migration of Huh-7. **l** Transwell analysis of the cell invasion of Huh-7. **p < 0.001, Student’s t test

To ask whether NQO1 was required for the oncogenic effect of CPSF6, we knocked down NQO1 expression in HL-7702 (the control) and CPSF6-overexpressing HL-7702 cells by shRNA, respectively (Fig. 6a). Although CPSF6 overexpression promoted cell proliferation, migration and invasion in HL-7702, this effect was almost depleted in NQO1 knockdown cells (Fig. 6b-f), suggesting that CPSF6 function is NQO1-dependent. Furthermore, we ectopically expressed NQO1 in CPSF6 knockdown Huh-7 cells (Fig. 6g), and found that although knockdown of CPSF6 inhibited cell proliferation, migration and invasion in Huh-7, restoring the expression of NQO1 obviously reversed these tumorigenic activities (Fig. 6h-l).

### CPSF6 regulates liver cell metabolism through NQO1

**Fig. 7:**
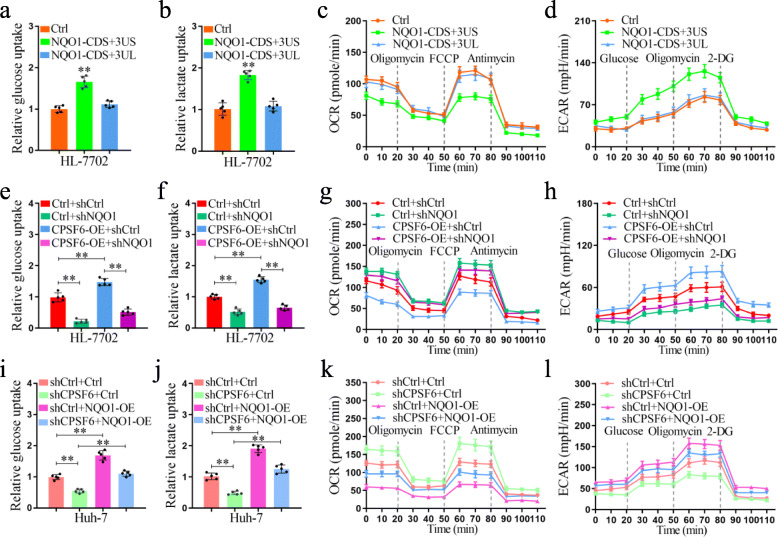
CPSF6 upregulates glycolysis and promotes aerobic glycolysis through NQO1. **a** Glucose uptake in control and NQO1-overexpressing HL-7702 cells. **b** Cellular lactate levels in control and NQO1-overexpressing HL-7702 cells. OCR (**c**) and ECAR (**d**) of control and NQO1- overexpressing HL-7702 cells measured by the Seahorse Bioscience XF96 analyzer. **e** Glucose uptake in control and CPSF6-overexpressing HL-7702 cells with or without NQO1 knockdown. **f** Cellular lactate levels in control and CPSF6-overexpressing HL-7702 cells with or without NQO1 knockdown. OCR (**g**) and ECAR (**h**) of control and CPSF6-overexpressing HL-7702 cells with or without NQO1 knockdown measured by the Seahorse Bioscience XF96 analyzer. **i** Glucose uptake in control and CPSF6 knockdown Huh-7 cells with or without overexpression of NQO1. **j** Cellular lactate levels in control and CPSF6 knockdown Huh-7 cells with or without overexpression of NQO1. OCR (**k**) and ECAR (**l**) of control and CPSF6 knockdown Huh-7 cells with or without overexpression of NQO1 measured by the Seahorse Bioscience XF96 analyzer. **p < 0.001, Student’s t test

It has been reported that NQO1 can regulate cell metabolism [42]. It is unclear whether the *NQO1* isoforms are involved in metabolism regulation in HCC. To address this issue, we tested the role of distinct *NQO1* isoforms in HL-7702 cells. We found the enforced expression of the *NQO1* isoform with short 3′UTR dramatically increased glucose uptake (Fig. 7a) and lactate production (Fig. 7b), when compared with that of vector control and ectopic expression of long 3′UTR isoform. In addition, the enforced expression of the short isoform dramatically decreased basal as well as maximum oxygen consumption rate (OCR) (Fig. 7c) but increased extracellular acidification rate (ECAR) (Fig. 7d), when compared with that of vector control and ectopic expression of long 3′UTR isoform.

Although overexpression of CPSF6 in HL-7702 dramatically increased glucose uptake, lactate production, ECAR, and decreased OCR (Fig. 7e-h), we found this trend was obviously reversed after NQO1 knockdown (Fig. 7e-h). Similar to NQO1 knockdown, CPSF6 knockdown cells also exhibited reduced glucose uptake and lactate production (Fig. 7i, j), increased basal as well as maximum OCR (Fig. 7k), and decreased ECAR (Fig. 7l). These observations suggest that CPSF6 promotes aerobic glycolysis and suppresses oxidative phosphorylation in liver cells through NQO1.

### NQO1 is upregulated in HCC and positively correlates with CPSF6 expression

**Fig. 8:**
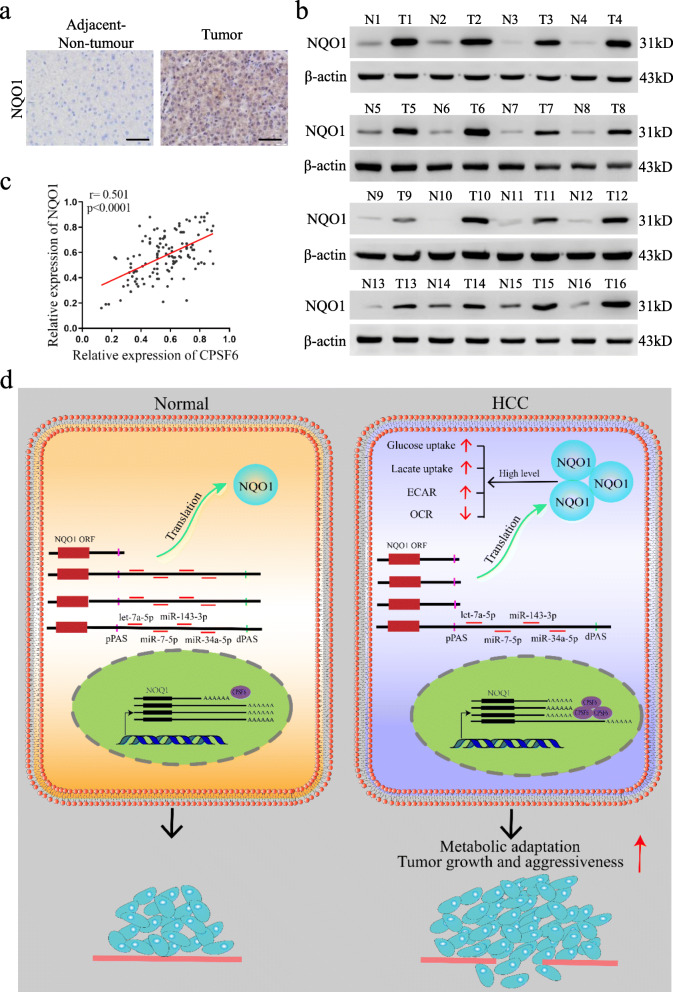
Expression of NQO1 in human HCC tissues. **a** IHC staining of NQO1 in surrounding non-tumor and HCC tissues. Scale bar, 50 μm. **b** Western blot analysis of NQO1 expression in 16 paired surrounding non-tumor (N) and HCC tumor (T) tissues. β-actin was used as internal control. **c** The correlation analysis between NQO1 expression and CPSF6 level in 124 HCC tissues (TNM, III/IV) with linear regression and pearson’s correlation significance (*P* < 0.0001, ANOVA test). **d** The proposed mechanism of CPSF6 in regulation of HCC progression

We lastly examined the NQO1 expression in both HCC and the paired non-tumor tissues. A total of 159 cases of HCC and paired non-tumor samples were collected for IHC assay. We observed the upregulated expression of NQO1 in tumor tissues when comparing with the paired non-tumor samples (Fig. 8a). This observation was confirmed by western blotting assay of another 36 patients (Fig. 8b and Supplementary Fig. S4). We next examined the expression relationship of NOQ1 and CPSF6. We observed a moderately positive correlation between CPSF6 and NQO1 in the 124 patients with stage III and IV (r = 0.501, Fig. 8c; Supplementary Table S1). These findings suggest NQO1 is upregulated in HCC and positively correlates with CPSF6 expression.

## Discussion

As an important mechanism in regulation of gene expression, APA is widespread across human genome. Dysregulation of APA is increasingly recognized as tumorigenesis driver [15]. APA is specified by a number of regulatory factors [7], among which polyadenylation factors have been intensively characterized. For example, CFIm25 causes 3′UTR shortening of a number of genes in glioblastoma cells and promotes tumor suppression [22]; however, CSTF2 in urothelial carcinoma of the bladder elicits 3′UTR shortening of *RAC1* and contributes to UCB pathogenesis [23]. These studies underline the necessity of APA factor characterization at different cellular context. CPSF6 has been recently reported to be of clinical relevance in breast cancer [32]. However, the role of CPSF6 in HCC is still largely unknown.

Some recent studies have reported the altered expression pattern of APA factors in HCC. For example, Ji and colleagues found CPSF7 is activated and regulates liver cancer growth and metastasis by targeting the WWP2/PTEN/AKT signaling pathway [43]. In contrast, we reported that the expression of NUDT21 is repressed, which contributes to hepatocellular carcinoma suppression [25]. In the present study we observed that CPSF6 is highly expressed in HCC tissues and associated with poor prognosis. We then performed a combination of in vitro and in vivo assays to understand the function of CPSF6. Our findings clearly showed that overexpression of CPSF6 significantly promoted the tumorigenic and metastatic activities either in vitro or in vivo. Our results suggest that CPSF6 exerts oncogenic effect in HCC.

Given CPSF6 as an APA factor per se, one of intriguing question was thus raised up, i.e., what mechanisms underlie the oncogenic effect of this CFIm subunit? We then investigated APA profiles under the conditions of high and low levels of CPSF6. Seven genes with altered 3’UTR were simultaneously found under both conditions, including *NQO1*.

NQO1 is a cytosolic flavoprotein that catalyzes the reduction of quinones and protects cells from oxidative stress through preventing the generation of free radicals [42]. Upregulation of NQO1 was frequently observed in various types of cancer, such as breast, prostate, lung and liver cancer [44–47]. Nevertheless, the regulatory mechanism of NQO1 expression has not well characterized. Our results showed that overexpression of CPSF6 caused the preferential proximal poly(A) site usage in the 3′UTR of *NQO1* and increased the level of NQO1 protein. The activation of CPSF6-induced *NQO1* isoform with short 3′UTR, at least partially due to the escape from miRNA repression, was confirmed by luciferase activity assays. In addition to CPSF6, it should be noted that another APA factor CstF-64 also modulates APA pattern of *NQO1* in HEK 293 cells [48], suggesting that the selective poly(A) site usage of *NQO1* is regulated in cellular context-dependent manner.

The Warburg effect is a general feature of glucose metabolism in cancer cells, which is intimately linked to the uncontrolled and continuing proliferation characteristic of cancer cells [49–51]. Numerous studies have documented a strong association between HCC and the metabolic alterations [36, 37, 52, 53]. Very recently, NQO1 has been reported to be involved in metabolic adaptation and affect glycolysis and glutaminolysis in HCC [54]. Our results showed that enforced expression of the *NQO1* isoform with short 3′UTR dramatically increased glucose uptake and lactate production. We further observed that such enforced expression dramatically decreased basal as well as maximum OCR, but increased ECAR. In HCC cells overexpression of NQO1 activated the PI3K/Akt and MAPK/ERK pathways and promoted metabolic adaptation [54]. Whether the CPSF6-induced high expression of NQO1 contributes to the activation of these pathways is unknown. In addition, the finding of CPSF6-associated metabolic adaptation was observed in the cell culture system, it remains unclear whether the similar scenario happens in vivo and requires further investigation.

## Conclusions

In summary, our study described that CPSF6 exerts oncogenic effect on HCC cells by promoting 3′UTR shortening of *NQO1* (Fig. 8d). To the best of our knowledge, this study, for the first time, suggested a link between APA and metabolism adaption in cancer. The functional and mechanistic results generated in our study might offer potential therapeutic targets for the treatment options against HCC.

## Supplementary Information

**Additional file 1 Supplementary Fig. S1.** The expression of CPSF6 in HCC. Western blot assay for the CPSF6 expression in 20 paired surrounding non-tumor (N) and HCC tumor (T) tissues. β-actin was used as internal control.

**Additional file 2 Supplementary Table S1.** Clinicopathologic correlation of CPSF6 expression in HCCs. The expression level of CPSF6 protein was detected by immunohistochemistry.

**Additional file 3 Supplementary Fig. S2.** CPSF6 accelerates the growth of HepG2 cells. **A,** CPSF6 knockdown efficiency in HepG2 cells with two CPSF6 specific shRNAs (shCPSF6–1 and shCPSF6–2) was examined by western blotting. β-actin was used as internal control. **B,** Proliferation curves of HepG2 cells with stable expression of CPSF6 shRNA or the control (shCtrl) were shown. **C,** The cell viability of CPSF6-silencing HepG2 cells and HepG2 cells (the control) was examined by MTT assay. **D,** Colony formation analysis of HepG2 cells. **p < 0.001, Student’s t test.

**Additional file 4 Supplementary Fig. S3.** CPSF6 promotes migration and invasion of HepG2 cells. **A,** Effects of CPSF6-silencing on HepG2 cell migration. **B,** Effects of CPSF6-silencing on HepG2 cell invasion. Scale bars, 100 μm. **p < 0.001, Student’s t test.

**Additional file 5 Supplementary Table S2.** The sequencing statistics of 3 T-seq data.

**Additional file 6 Supplementary Table S3.** The 3′UTR shortened genes in CPSF6-OE HL-7702 cells and the 3′UTR lengthened genes in CPSF6-KD Huh7 cells.

**Additional file 7 Supplementary Table S4.** The list of genes with altered 3’UTR shared by HL-7702 and Huh7 cells.

**Additional file 8 Supplementary Fig. S4.** The expression of NQO1 in HCC. Western blot assay for the NQO1 expression in 20 paired surrounding non-tumor (N) and HCC tumor (T) tissues. β-actin was used as internal control.

## Data Availability

All data generated or analysed during this study are included in this published article [and its supplementary information files].

## Abbreviations

HCC: Hepatocellular carcinoma
IHC: Immunological histological chemistry
TCGA: The cancer genome atlas
OS: Overall survival
PFS: Progress-free survival
OE: Overexpression
KD: Knockdown
miRNA: MicroRNA
NS: No significant difference
HE staining: Hematoxylin-eosin staining
ECAR: extracellular acidification rate
OCR: Oxygen consumption rate
TNM: Tumor node metastasis
