# Supplementary figures and images for: CPSF6 links alternative polyadenylation to metabolism adaption in hepatocellular carcinoma progression
Source: J Exp Clin Cancer Res. 2021 Mar 1;40:85. doi: 10.1186/s13046-021-01884-z (PMC7923339; doi:10.1186/s13046-021-01884-z)

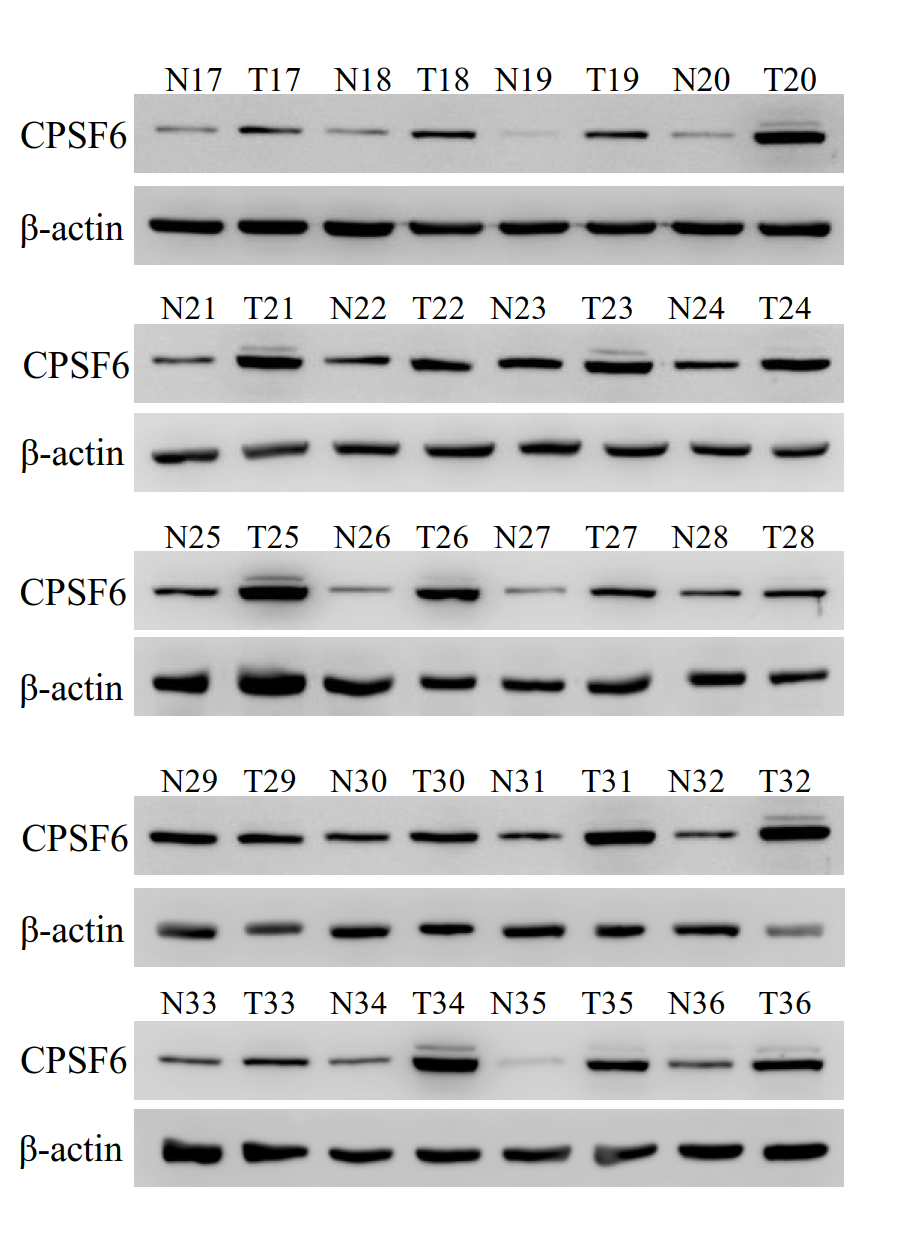

Supplement: Supplementary file 1 — Additional file 1 Supplementary Fig. S1. The expression of CPSF6 in HCC. Western blot assay for the CPSF6 expression in 20 paired surrounding non-tumor (N) and HCC tumor (T) tissues. β-actin was used as internal control. [file 13046_2021_1884_MOESM1_ESM.tif]

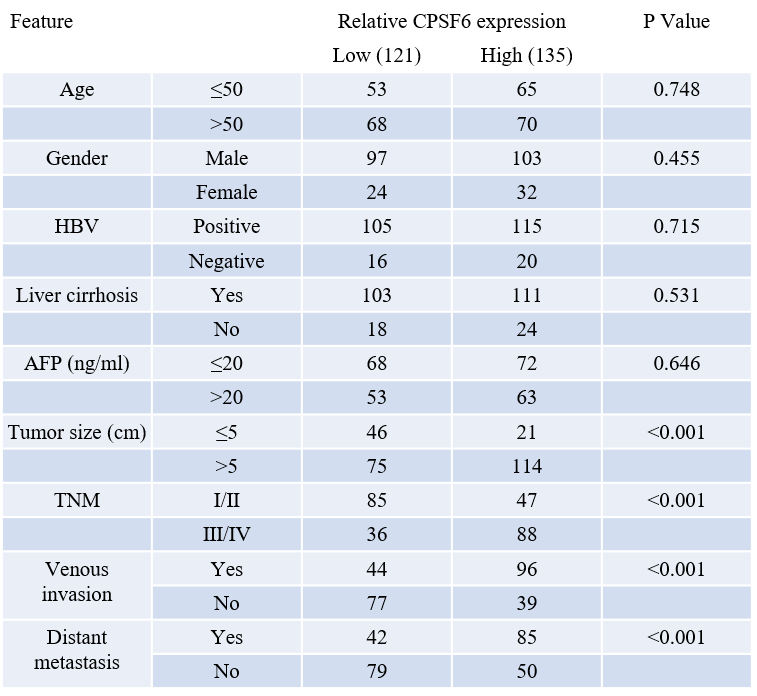


Table S1. Clinicopathologic correlation of CPSF6 expression in HCCs

Supplement: Supplementary file 2 — Additional file 2 Supplementary Table S1. Clinicopathologic correlation of CPSF6 expression in HCCs. The expression level of CPSF6 protein was detected by immunohistochemistry. [file 13046_2021_1884_MOESM2_ESM.docx]

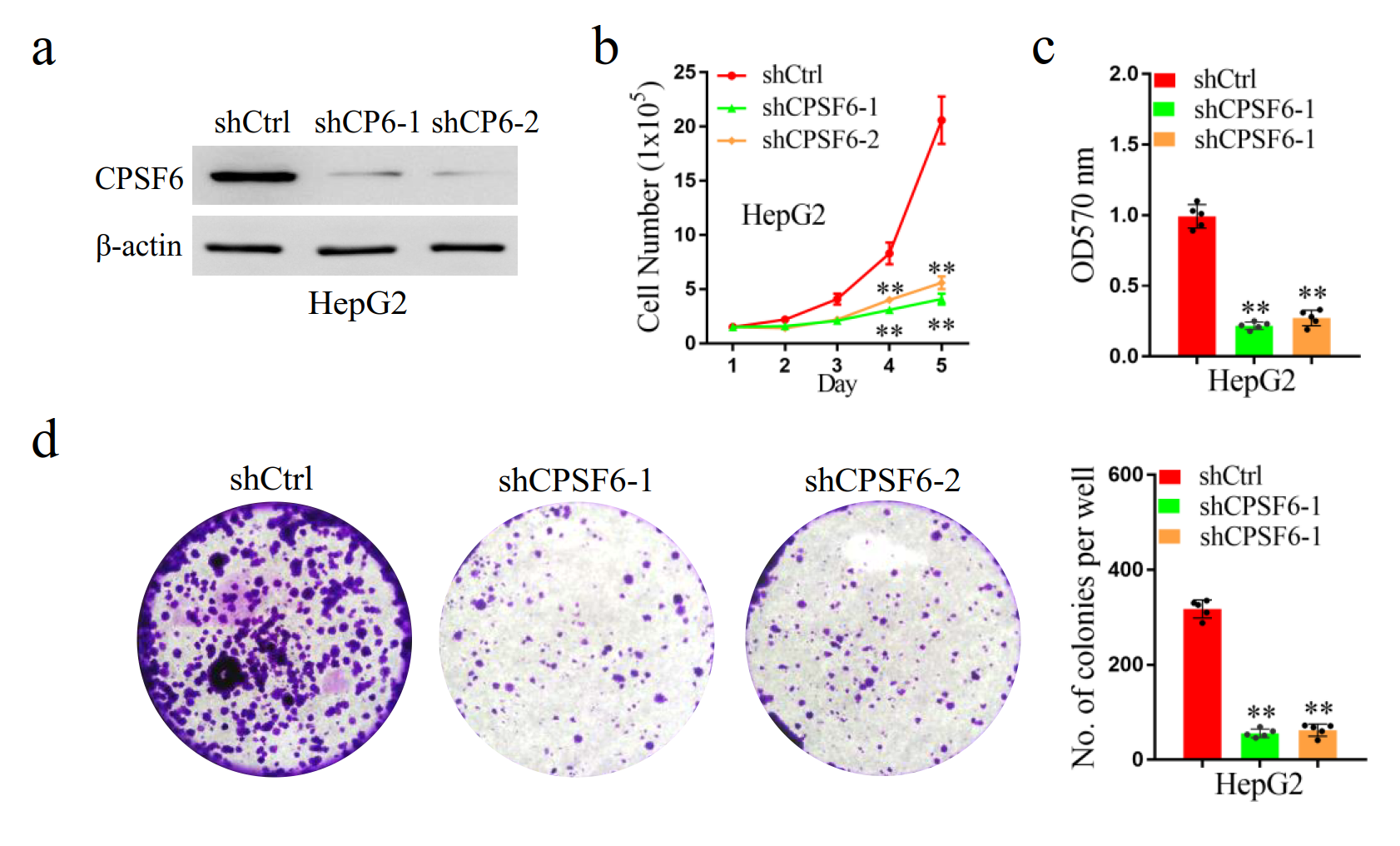

Supplement: Supplementary file 3 — Additional file 3 Supplementary Fig. S2. CPSF6 accelerates the growth of HepG2 cells. A, CPSF6 knockdown efficiency in HepG2 cells with two CPSF6 specific shRNAs (shCPSF6–1 and shCPSF6–2) was examined by western blotting. β-actin was used as internal control. B, Proliferation curves of HepG2 cells with stable expression of CPSF6 shRNA or the control (shCtrl) were shown. C, The cell viability of CPSF6-silencing HepG2 cells and HepG2 cells (the control) was examined by MTT assay. D, Colony formation analysis of HepG2 cells. **p < 0.001, Student’s t test. [file 13046_2021_1884_MOESM3_ESM.tif]

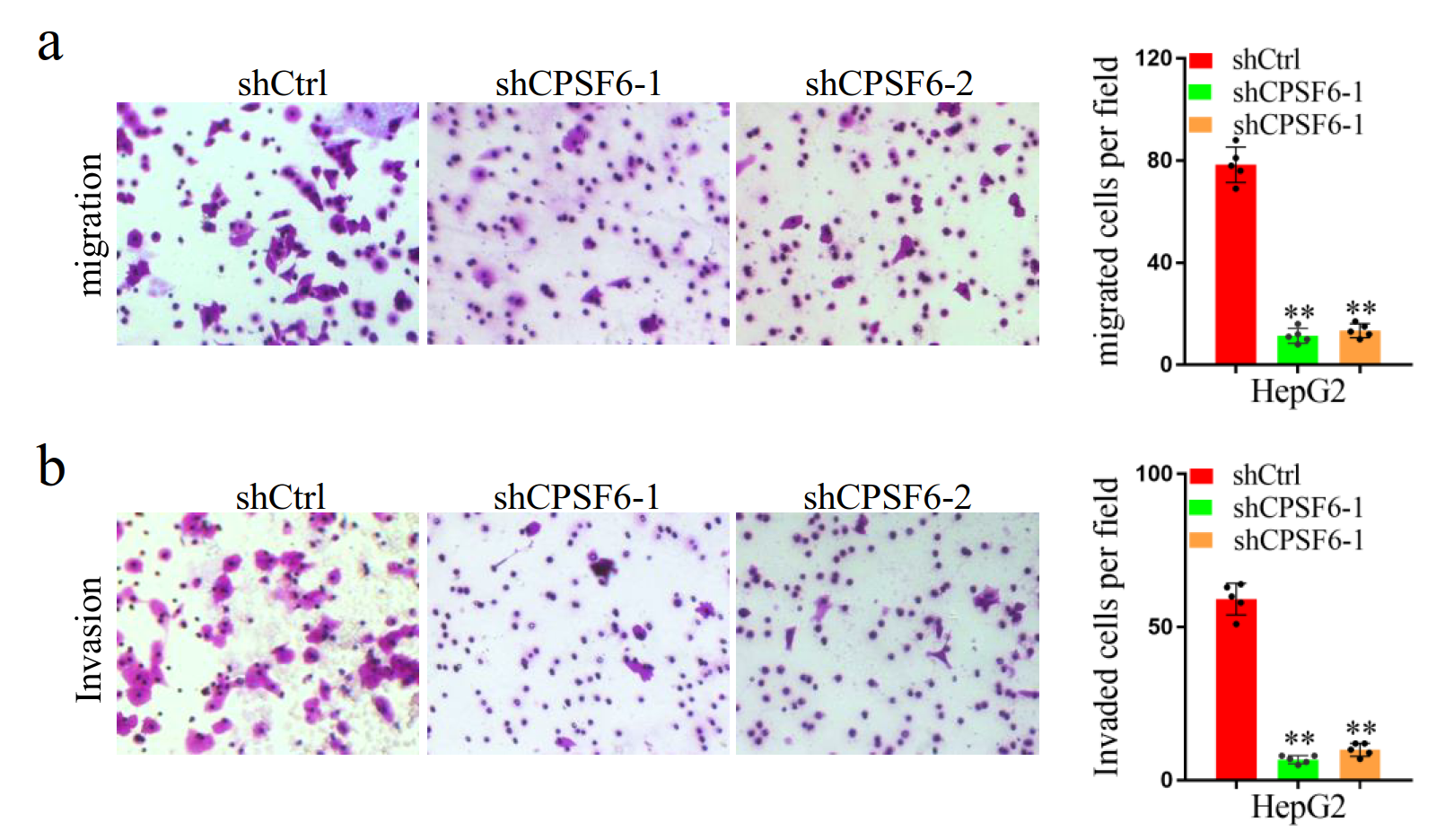

Supplement: Supplementary file 4 — Additional file 4 Supplementary Fig. S3. CPSF6 promotes migration and invasion of HepG2 cells. A, Effects of CPSF6-silencing on HepG2 cell migration. B, Effects of CPSF6-silencing on HepG2 cell invasion. Scale bars, 100 μm. **p < 0.001, Student’s t test. [file 13046_2021_1884_MOESM4_ESM.tif]

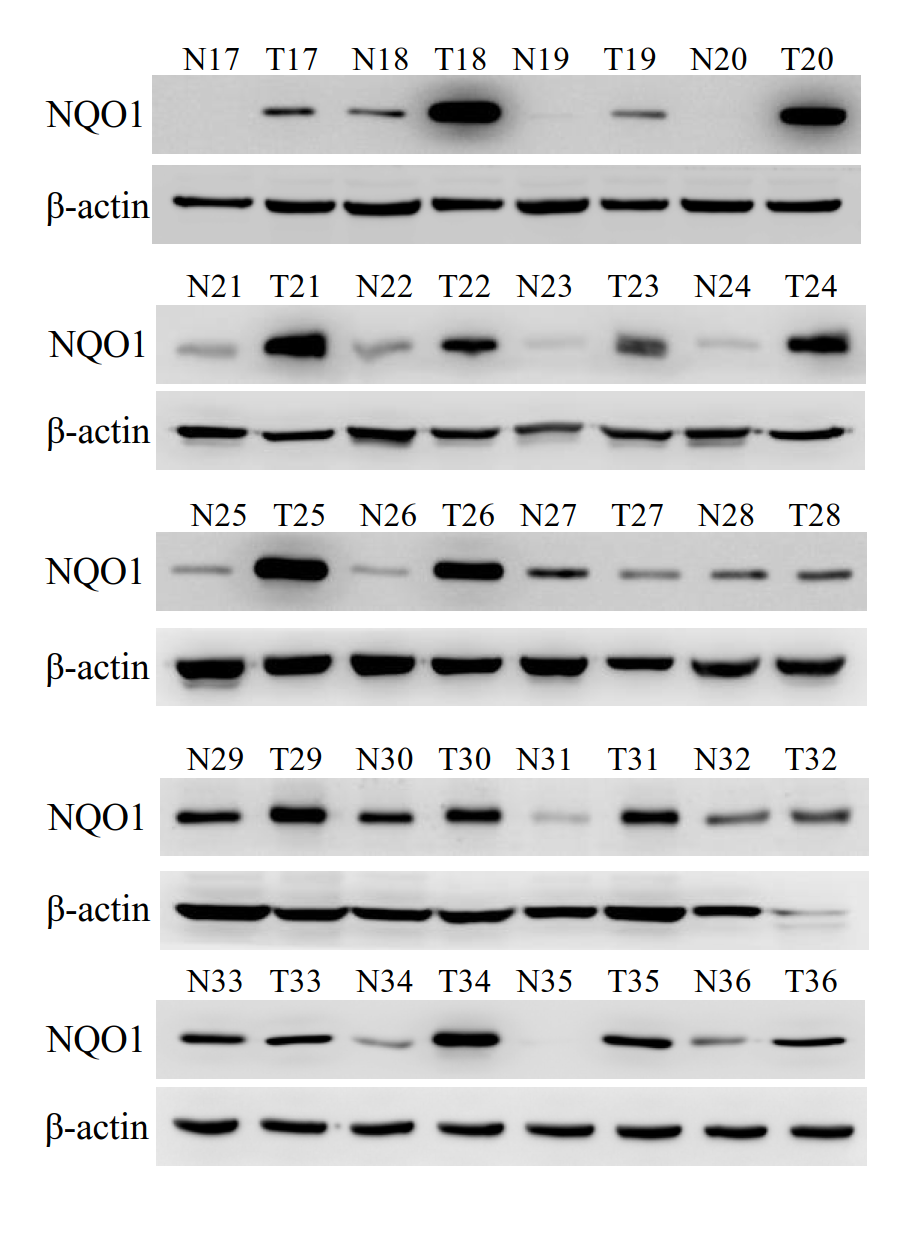

Supplement: Supplementary file 8 — Additional file 8 Supplementary Fig. S4. The expression of NQO1 in HCC. Western blot assay for the NQO1 expression in 20 paired surrounding non-tumor (N) and HCC tumor (T) tissues. β-actin was used as internal control. [file 13046_2021_1884_MOESM8_ESM.tif]
